# The risk factors for deep venous thrombosis in critically ill older adult patients: a subgroup analysis of a prospective, multicenter, observational study

**DOI:** 10.1186/s12877-022-03599-y

**Published:** 2022-12-19

**Authors:** Li Li, Junhai Zhen, Liquan Huang, Jia Zhou, Lina Yao, Lingen Xu, Weimin Zhang, Gensheng Zhang, Qijiang Chen, Bihuan Cheng, Shijin Gong, Guolong Cai, Ronglin Jiang, Jing Yan

**Affiliations:** 1 Department of Critical Care Medicine, Zhejiang Hospital, 12 Lingyin Road, Hangzhou, 310013 China; 2 Department of Critical Care Medicine, Zhejiang Provincial Hospital of Chinese Medicine, 54 Youdian Road, Hangzhou, 310013 China; 3 Department of Critical Care Medicine, Ningbo Yinzhou People’s Hospital, 58 Zhoumeng North Road, Yinzhou, Ningbo City, 315100 China; 4 Department of Critical Care Medicine, Xinchang Hospital of Traditional Chinese Medicine, 188 Jiufeng Road, Xinchang, Shaoxing City, 312500 China; 5 Department of Critical Care Medicine, Dongyang People’s Hospital, 60 Wuning West Road, Dongyang, Jinhua City, 322100 China; 6 Department of Critical Care Medicine, The Second Affiliated Hospital Zhejiang University School of Medicine, 88 Jiefang Road, Hangzhou, 310009 China; 7 Department of Critical Care Medicine, Ninghai First Hospital, 142 Taoyuan Middle Road, Ninghai, Ningbo City, 315600 China; 8 Department of Critical Care Medicine, The 2nd School of Medicine, Wenzhou Medical University, 109 West Xueyuan Road, Wenzhou, 325027 China

**Keywords:** Older adult patients, Intensive care unit, Deep venous thrombosis, Venous thromboembolism, Risk factor, China

## Abstract

**Background:**

Older adult patients mainly suffer from multiple comorbidities and are at a higher risk of deep venous thrombosis (DVT) during their stay in the intensive care unit (ICU) than younger adult patients. This study aimed to analyze the risk factors for DVT in critically ill older adult patients.

**Methods:**

This was a subgroup analysis of a prospective, multicenter, observational study of patients who were admitted to the ICU of 54 hospitals in Zhejiang Province from September 2019 to January 2020 (ChiCTR1900024956). Patients aged > 60 years old on ICU admission were included. The primary outcome was DVT during the ICU stay. The secondary outcomes were the 28- and 60-day survival rates, duration of stay in ICU, length of hospitalization, pulmonary embolism, incidence of bleeding events, and 60-day coagulopathy.

**Results:**

A total of 650 patients were finally included. DVT occurred in 44 (2.3%) patients. The multivariable logistic regression analysis showed that age (≥75 vs 60-74 years old, odds ratio (OR) = 2.091, 95% confidence interval (CI): 1.308-2.846, *P* = 0.001), the use of analgesic/sedative/muscarinic drugs (OR = 2.451, 95%CI: 1.814-7.385, *P* = 0.011), D-dimer level (OR = 1.937, 95%CI: 1.511-3.063, *P* = 0.006), high Caprini risk score (OR = 2.862, 95%CI: 1.321-2.318, *P* = 0.039), basic prophylaxis (OR = 0.111, 95%CI: 0.029-0.430, *P* = 0.001), and physical prophylaxis (OR = 0.322, 95%CI: 0.109-0.954, *P* = 0.041) were independently associated with DVT. There were no significant differences in 28- and 60-day survival rates, duration of stay in ICU, total length of hospitalization, 60-day pulmonary embolism, and coagulation dysfunction between the two groups, while the DVT group had a higher incidence of bleeding events (2.6% vs. 8.9%, *P* < 0.001).

**Conclusion:**

In critically ill older adult patients, basic prophylaxis and physical prophylaxis were found as independent protective factors for DVT. Age (≥75 years old), the use of analgesic/sedative/muscarinic drugs, D-dimer level, and high Caprini risk score were noted as independent risk factors for DVT.

**Trial registration:**

Chinese Clinical Trial Registry (ChiCTR1900024956).URL: http://www.chictr.org.cn/listbycreater.aspx.

## Background

Deep venous thrombosis (DVT) is a type of venous thrombosis involving the formation of a blood clot in a deep vein, most commonly in the legs or pelvis [1–3]. Patients who develop DVT commonly have risk factors, such as active cancer, trauma, major surgery, hospitalization, immobilization, pregnancy, or oral contraceptive use. An unprovoked DVT can be idiopathic or result from inherited or acquired hypercoagulable states, such as cancer and pregnancy [1–3]. The number of adults with venous thromboembolic events (such as DVT) in the United States is projected to more than double by 2050, and it was reported that the annual prevalence of venous thromboembolic events increased with age [1]. Patients hospitalized at intensive care units (ICUs) are at an even higher risk of DVT, mainly due to their clinical presentation and factors associated with an ICU admission, such as prolonged immobility, sedation, and neuromuscular blockade to facilitate ventilation. To date, few studies focused on DVT in older adult patients, especially in critically ill older adult patients. Engbers et al. found the annual incidence of venous thromboembolic events (such as DVT) in older adult patients to be about eight times higher than that in patients under 50 years old. Huang et al. reported the incidence of DVT sharply increased among those older than 75 years old. Some studies demonstrated that the prevalence of DVT during ICU stay was about 7.3% [4–6].

Reduced blood flow caused by prolonged periods of inactivity, especially in older adult subjects, long hospitalizations due to illness, pregnancy, and long-distance travel with limited movements, such as air travel, are associated with increased risk of DVT. Similarly, individuals with increased levels of clotting factors in the circulation resulting from diseases, medications, or inherited traits, have an increased risk of DVT. The risk of DVT is associated with an elevation in the blood fibrinogen level (hyperfibrinogenemia), as well as abnormal fibrin clot structure and function. Compared with individuals with normal circulating fibrinogen levels, individuals with higher fibrinogen levels (> 4 g/L) were 2-fold more disposed to experience DVT, and this was significant in older patients [1–3]. According to the above-mentioned risk factors, it can be inferred that the majority of patients in the ICU are particularly vulnerable to DVT because of immobility, critical conditions (e.g., trauma or surgery), multiple invasive procedures, and inflammation [7, 8]. It is noteworthy that a DVT event in vulnerable patients with a history of trauma or surgery can lead to poor outcomes [7–9].

Furthermore, older adult patients mainly suffer from multiple comorbidities [10] that can directly or indirectly increase the risk of DVT [1–3]. To date, although numerous studies have explored the risk factors for DVT, few studies have specifically concentrated on the risk factors for DVT in older adult patients. There is a need for a better understanding of the risk factors for DVT in older adult patients [11]. Length of stay in the ICU and older age are two important risk factors for DVT [7, 8], while further research needs to be conducted to clarify the exact risk factors for DVT in critically ill older adult patients. A recent study showed that the risk factors for venous thromboembolism in critically ill older adult patients were sex (male), bedridden for > 72 h, pneumonia, history of DVT, diabetes, coronary heart disease, glucocorticoids, PaO_2_ level, mechanical ventilation, continuous renal replacement therapy (CRRT), prothrombin time (PT), international normalized ratio (INR), and D-dimer level [12], while it was a retrospective study with a risk of bias. Therefore, prospective cohort studies are necessary to identify the risk factors of DVT in critically ill older adult patients.

Hence, the present study, based on prospectively acquired data, aimed to analyze the risk factors for DVT in critically ill older adult patients. The results could assist clinicians in the better management of critically ill older adult patients and provide a more personalized therapeutic management.

## Methods

### Study design and patients

In the present post hoc subgroup analysis of a prospective, multicenter, observational study, patients who were hospitalized in the ICU of 54 hospitals in Zhejiang Province (China) from September 16, 2019, to January 16, 2020, were enrolled [13]. The approval of the study was carried out by the Medical Ethics Committee of Zhejiang Hospital (2019-24 K), and all patients or their family members signed the written informed consent form prior to enrollment. Registration of the study in the Chinese Clinical Trial Registry (ChiCTR1900024956) was performed, and it was followed in accordance with the tenets of the Declaration of Helsinki and the Good Clinical Practice.

The inclusion criterion was set to include ICU patients aged > 60 years old. The exclusion criteria were as follows: 1) Diagnosis of DVT or pulmonary embolism prior to ICU admission, 2) Length of stay in ICU < 48 h, 3) Occurrence of death within 48 h after admission, or 4) Patients with advanced cancer. The diagnosis of DVT was performed according to the criteria presented by the Chinese guidelines for the diagnosis and treatment of deep vein thrombosis (3rd edition) [14].

### Assessment of the risk of DVT

The risk of DVT was evaluated through the Wells DVT risk assessment scale, Caprini risk score scale, and Padua risk score scale. The first assessment was performed within 24 or at 24-48 h after admission, followed by reassessment after changing patients’ conditions on a regular basis. A change in the condition was attributed to the reduction of blood pressure to < 90/60 mmHg or a higher than 30% reduction, PO_2_ < 60 mmHg, or the necessity of undergoing invasive or emergency surgery. To routinely perform reassessment, the frequencies of once a month, once a week, twice a week, or daily were considered.

### Prophylaxis of DVT

According to the outcomes of the risk assessment, the patients were given basic, physical, or drug prophylaxis for DVT. Using the 2018 edition of the “Guidelines for the Diagnosis, Treatment, and Prevention of Pulmonary Thromboembolism” [15] and the 2020 edition of the “Chinese Expert Consensus on Mechanical Prevention of Venous Thromboembolism”, an effort was made to carry out prophylaxis [16]. Basic prophylaxis included blood lipid and glucose control, raising the affected limb, and early functional training. For patients who are at a high risk of DVT and a low risk of hemorrhage, drug prophylaxis included unfractionated heparin (UFH), low-molecular-weight heparin (LMWH), fondaparinux, new oral anticoagulants, and vitamin K antagonists. For patients who were at a high risk of DVT, while with dominancy of active hemorrhage or risk of hemorrhage was obvious, we attempted to use physical prophylaxis, such as intermittent pneumatic compression (IPC, > 18 h/day), graduated compression stockings (GCS, worn in the whole day), and venous foot pumps (VFPs, > 18 h/day).

### Diagnosis and treatment of DVT

We, in the present study, diagnosed and treated DVT on the basis of the 2017 “Guidelines for the Diagnosis and Treatment of Deep Vein Thrombosis (Third Edition)” presented by the Chinese Society of Vascular Surgery of the Chinese Medical Association [14] and the 2018 Chinese Thoracic Society “Guidelines for the Diagnosis, Treatment, and Prevention of Pulmonary Thromboembolism” [15]. One of the UFH, LMWH, and vitamin K antagonists (e.g., warfarin) was used to treat patients. According to a patient’s clinical conditions, the therapy was changed. The initial dose of UFH was 80-100 U/kg intravenously, followed by 10-20 U/kg/h intravenously. On the basis of the activated partial thromboplastin time (APTT), the mentioned dose was adjusted every 4-6 h, leading to the extension of APTT to 1.5-2.5 times the normal control value. The dose of LMWH was subcutaneously set to 100 U/kg once every 12 h. For patients aged > 75 years old and were at a high hemorrhage risk, the initial dose of warfarin was 3.0-5.0 mg/d or 2.5-3.0 mg. Besides, streptokinase, urokinase, and recombinant human tissue plasminogen activator (rt-PA) were utilized as thrombolysis therapy, and each one was separately used. For streptokinase, the loading dose was 250,000 U intravenously for 30 min, followed by a maintenance intravenous infusion of 100,000 U/h for 12-24 h. For urokinase, the loading dose was 4400 U/kg intravenously for 10 min, followed by 2200 U/kg/h with a continuous intravenous drip for 12 h. For rt-PA, the dose was 50 mg, and infusion was continuously performed for 2 h.

### Data collection and follow-up

The patient’s baseline characteristics were collected, including age, sex, height, weight, vital signs, vasoactive drug use, deep vein catheterization, Acute Physiology and Chronic Health Evaluation II (APACHE II) scores, laboratory tests, and vascular Doppler examinations. All laboratory examinations were performed within 24 h after admission. The follow-up of patients was performed only during their hospitalization. The bleeding events recorded were after DVT was diagnosed. For all risk scores (e.g., APACHE II, Caprini, and Padua), the worst value was used for analysis.

### Outcomes

In the current study, DVT occurrence was considered the primary outcome during patients’ stay in ICU. Additionally, 28- and 60-day survival rates, length of stay in ICU, the total length of hospitalization, pulmonary embolism, incidence of hemorrhage events, and coagulopathy within 60 days were defined as secondary outcomes. The hemorrhage events included intracerebral hemorrhage (ICH) and gastrointestinal hemorrhage. ICH was proven by CT in the hospital regardless of whether the condition of the patient changed. Gastrointestinal hemorrhage included hematemesis and hematochezia. The diagnosis of coagulopathy was based on activated partial thromboplastin time (APTT), prothrombin time (PT), thrombin time (TT), international normalized ratio (INR), platelet count, fibrinogen levels, disseminated intravascular coagulation (DIC) score of 1-4 (non-overt DIC) or ≥ 5 (overt DIC), or abnormalities in clotting amplitude and clot lysis in whole blood visco-elastic tests. The clinical manifestations often include different degrees of bleeding or coagulation.

### Statistical analysis

This study had a case-control design. The Shapiro-Wilk test was used to determine whether the continuous variables followed the normal distribution. The normally distributed continuous variables were presented as mean ± standard deviation, and their comparison was made using Student’s t-test; the abnormally distributed continuous variables were presented as median (range), and their comparison was made by the Mann-Whitney *U*-test. The expression of categorical variables was in the form of n (%), and the chi-square test or Fisher’s exact test was used for comparisons. The influences of confounders were minimized using propensity score matching (PSM). The matching of the DVT and non-DVT groups was conducted at a ratio of 1:4 (random sampling method; clamp value of 0.1) according to the patient source (i.e., specific department before ICU admission) and reason for ICU admission. Factors influencing the DVT were analyzed by the logistic regression analysis. The variables with *P*-values < 0.05 in the univariable analyses were included in the multivariable logistic regression analysis. Odds ratio (ORs) > 1 indicated that the factor was associated with the presence of DVT, while ORs < 1 indicated that the factor was associated with the absence of DVT. The level of statistical significance was set to *P* < 0.05. Using SPSS 26.0 software (IBM, Armonk, NY, USA), the statistical analysis was carried out.

## Results

### Characteristics of the patients

**Fig. 1:**
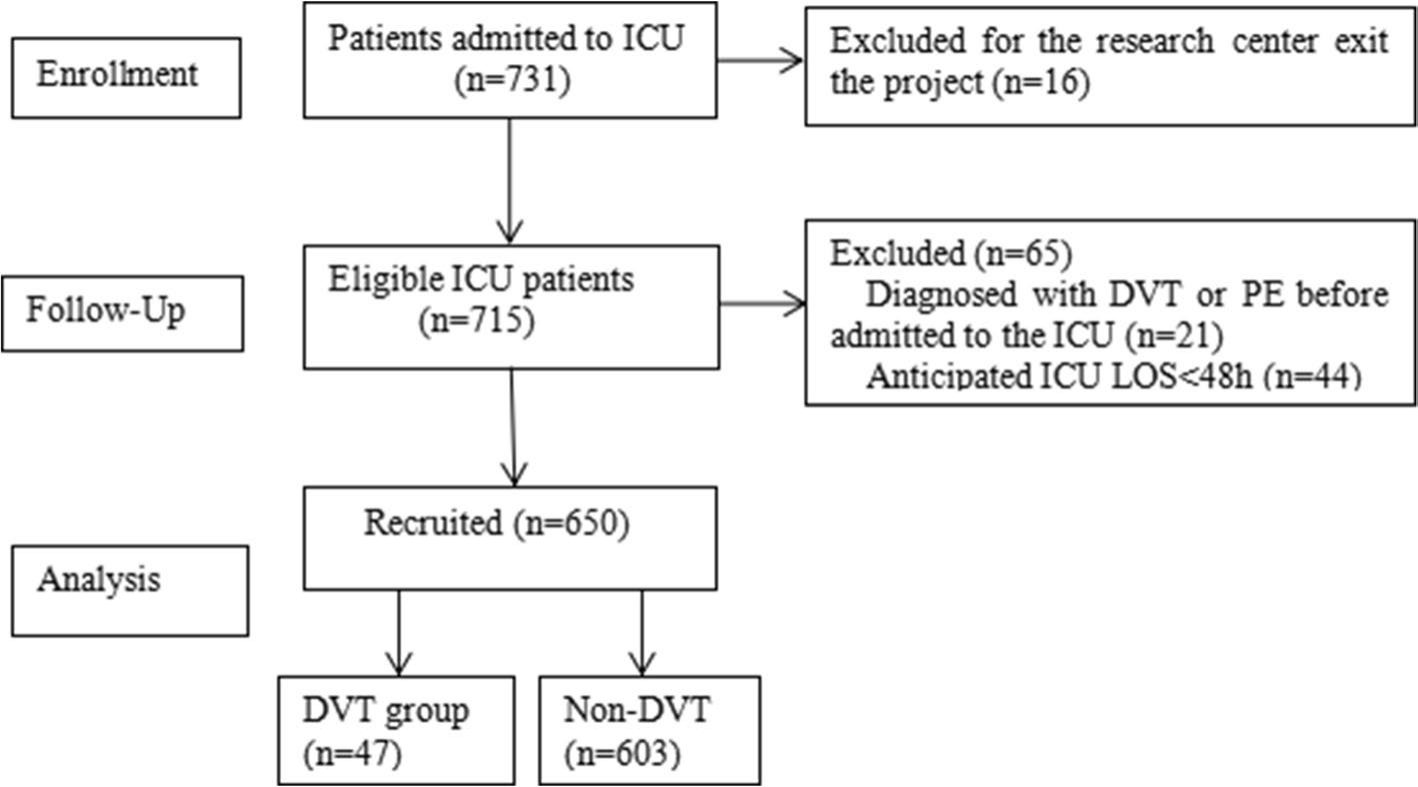
Study flowchart. ICU: intensive care unit; DVT: deep venous thrombosis; LOS: length of stay

Figure 1 shows the study flowchart. A total of 731 patients aged ≥60 years were admitted to ICU during the study period, of whom 21 cases were diagnosed with DVT or pulmonary embolism prior to ICU admission, 44 were expected to be admitted to ICU for < 48 h, and 16 cases withdrew their consent, resulting in the inclusion of 650 patients in the present analysis. DVT occurred in 47 (7.2%) patients during their stay in ICU. After PSM, there were statistically significant differences in age, APTT, D-dimer levels, the incidence of chronic obstructive pulmonary disease (COPD), the incidence of cerebrovascular disease (CVD), history of surgical procedures, use of analgesic/sedative/muscarinic drugs (all *P* < 0.05, Table 1).

**Table 1:** Comparison of baseline data of the two groups before and after PSM

Variables	Total (*n* = 650)	Before PSM				After PSM			
		DVT (*n* = 47)	Non-DVT (*n* = 603)	*P*	Std. Mean Diff	DVT (*n* = 47)	Non-DVT (*n* = 169)	*P*	Std. Mean Diff
Age (years)	76.3 (69.0–84.0)	82.0 (72.0–86.0)	72.0 (66.0–82.0)	0.020		82.0 (72.0–86.0)	75.0 (67.0–82.0)	0.008	
60–74	278 (42.8%)	13 (27.7%)	265 (43.9%)	0.030	0.974	13 (27.7%)	80 (47.3%)	0.016	0.881
≥ 75	323 (49.7%)	28 (59.6%)	295 (48.9%)		0.497	28 (59.6%)	78 (46.2%)		0.492
BMI (kg/m^2^)	22.7 (20.1–23.9)	22.4 (20.9–23.6)	22.2 (20.0–24.0)	0.483	1.402	22.4 (20.9–23.6)	22.4 (20.2–23.4)	0.548	0.896
Sex (male)	432 (66.5%)	33 (70.2%)	399 (66.2%)	0.572	0.472	33 (70.2%)	108 (63.9%)	0.422	0.471
Underlying diseases									
COPD	140 (21.5%)	12 (25.5%)	128 (21.2%)	0.003	0.411	12 (25.5%)	33 (19.5%)	0.012	0.281
Hypertension	379 (58.3%)	23 (48.9%)	356 (59.0%)	0.176	0.494	23 (48.9%)	106 (62.7%)	0.088	0.502
Diabetes	143 (22.0%)	10 (21.3%)	133 (22.1%)	0.901	0.414	10 (21.3%)	43 (25.4%)	0.557	0.406
Cardiogenic diseases^a^	206 (31.7%)	10 (21.3%)	196 (32.5%)	0.111	0.465	10 (21.3%)	55 (32.5%)	0.136	0.448
Renal system diseases	58 (8.9%)	2 (4.3%)	56 (9.3%)	0.421	0.283	2 (4.3%)	18 (10.7%)	0.258	0.188
Gastrointestinal diseases	26 (4.0%)	0	26 (4.3%)	0.247	0.196	0	12 (7.1%)	0.073	0.000
Peripheral vascular lesions	19 (2.9%)	0	19 (3.1%)	0.387	0.169	0	3 (1.8%)	> 0.999	0.000
Long-term indwelling catheterization	57 (8.7%)	4 (8.5%)	53 (8.8%)	> 0.999	0.281	4 (8.5%)	28 (16.6%)	0.169	0.183
Cerebrovascular diseases^b^	141 (21.7%)	14 (29.8%)	127 (21.1%)	0.026	0.411	14 (29.8%)	40 (23.7%)	0.022	0.305
Malignant tumors	77 (11.8%)	4 (8.5%)	73 (12.1%)	0.463	0.323	4 (8.5%)	23 (13.6%)	0.350	0.305
Surgical history	149 (22.9%)	7 (14.9%)	142 (23.5%)	0.174	0.421	7 (14.9%)	54 (32.0%)	0.022	0.406
Sepsis	87 (13.4%)	15 (31.9%)	72 (11.9%)	0.566	0.342	15 (31.9%)	14 (8.3%)	0.571	0.254
Septic shock	65 (10.0%)	7 (14.9%)	63 (10.4%)	0.214	0.342	7 (14.9%)	10 (5.9%)	> 0.999	0.130
AKI	109 (16.8%)	6 (12.8%)	103 (17.1%)	0.508	0.374	6 (12.8%)	23 (13.6%)	0.953	0.363
Patient source									
Emergency room	308 (47.4%)	26 (55.3%)	282 (46.8%)	0.258	0.500	26 (55.3%)	84 (49.7%)	0.496	0.504
General ward	223 (34.3%)	7 (14.9%)	216 (35.8%)	0.004	0.475	7 (14.9%)	36 (21.3%)	0.330	0.418
Operating room	96 (14.8%)	12 (25.5%)	84 (13.9%)	0.031	0.355	12 (25.5%)	38 (22.5%)	0.661	0.457
Others^c^	23 (3.5%)	2 (4.3%)	21 (3.5%)	0.679	0.185	2 (4.3%)	11 (6.5%)	0.738	0.130
Reasons for admission to ICU									
Internal diseases	447 (68.8%)	18 (38.3%)	429 (71.1%)	< 0.001	0.465	18 (38.3%)	80 (47.3%)	0.271	0.247
Surgical diseases	161 (24.8%)	26 (55.3%)	135 (22.4%)	< 0.001	0.432	26 (55.3%)	87 (51.5%)	0.641	0.098
Traumatic injuries	37 (5.7%)	3 (6.4%)	34 (5.6%)	0.744	0.238	3 (6.4%)	2 (1.2%)	0.070	0.281
Others^d^	5 (0.8%)	0	5 (0.8%)	> 0.999	0.087			–	–
APACHE II	21.0 (14.0–25.0)	17.0 (12.0–26.0)	20.0 (10.0–24.0)	0.148	7.664	17.0 (12.0–26.0)	19.0 (13.0–25.0)	0.241	6.813
Biochemical indexes									
WBC (×10^9^/L)	12.2 (7.8–14.5)	11.2 (7.9–14.0)	10.6 (7.8–14.6)	0.862	1.074	11.2 (7.9–14.0)	10.1 (7.2–14.0)	0.407	1.045
Lactate (mmol/L)	2.6 (1.3–3.1)	1.8 (1.3–2.8)	1.9 (1.3–3.2)	0.641	2.320	1.8 (1.3–2.8)	1.8 (1.3–3.2)	0.613	1.492
CRP (mg/L)	71.5 (15.6–108.2)	75.5 (17.5–142.4)	48.5 (15.3–105.1)	0.138	1.295	75.5 (17.5–142.4)	42.5 (13.4–86.4)	0.067	1.789
APTT (s)	36.3 (28.5–40.5)	37.0 (32.5–44.4)	33.3 (28.3–40.2)	0.008	3.316	37.0 (32.5–44.4)	32.2 (28.0–38.8)	0.002	1.097
INR	1.3 (1.0–1.3)	1.2 (1.04–1.32	1.1 (1.0–1.3)	0.305	1.471	1.2 (1.04–1.32)	1.1 (1.01–1.32)	0.189	0.784
D-dimer (mg/dl)	4.8 (1.0–5.5)	10.4 (6.0–16.7)	2.3 (1.0–4.7)	< 0.001	2.809	10.4 (6.0–16.7)	2.5 (0.9–5.3)	< 0.001	1.085
Treatments, n (%)									
Deep vein catheterization	367 (56.5%)	30 (63.8%)	337 (55.9%)	0.359	0.496	30 (63.8%)	119 (70.4%)	0.498	0.464
Mechanical ventilation	446 (68.6%)	34 (72.3%)	412 (68.3%)	0.849	0.464	34 (72.3%)	129 (76.3%)	0.574	0.393
Analgesic, sedative drugs or muscle relaxants	390 (60.0%)	35 (74.5%)	355 (58.9%)	0.036	0.490	35 (74.5%)	109 (64.5%)	0.047	0.393
CRRT	62 (9.5%)	2 (4.3%)	60 (10.0%)	0.298	0.294	2 (4.3%)	10 (5.9%)	> 0.999	0.222

*PSM* Propensity score matching, *BMI* Body mass index, *COPD* Chronic obstructive pulmonary disease, *AKI* Acute kidney injury, *ICU* Intensive care unit, *APACHE II* Acute Physiology and Chronic Health Evaluation II, *WBC* White blood cells, *CRP* C-reactive protein, *APTT* Activated partial thromboplastin time, *INR* International normalized ratio, *CRRT* Continuous renal replacement therapy

^a^Including coronary artery disease, arrhythmia, and heart failure

^b^Including ischemic stroke and cerebral hemorrhage disease

^c^Including other ICU, inpatient beds, outpatient clinics

^d^Including various types of drug poisoning

After PSM, compared with the non-DVT group, the DVT group showed a lower frequency of assessment within 24 h (44.7% vs. 65.1%, *P* = 0.011), higher frequency of assessment at 24-48 h after admission (46.8% vs. 31.3%, *P* = 0.049), a higher frequency of DVT assessment when condition changed (38.3% vs. 3.0%, *P* < 0.001), lower use of basic prophylaxis (55.3% vs. 93.5%, *P* < 0.001), and lower use of physical prophylaxis (40.4% vs. 85.2%, *P* < 0.001) (Table 2).

**Table 2:** DVT risk assessment and prevention of the two groups before and after PSM

Variables	Total (*n* = 650)	Before PSM			After PSM		
		DVT (*n* = 47)	Non-DVT (*n* = 603)	*P*	DVT (*n* = 47)	Non-DVT (*n* = 169)	*P*
DVT risk assessment							
Padua low risk	33 (5.1%)	1 (2.2%)	32 (5.3%)	0.502	1 (2.2%)	4 (2.3%)	> 0.999
Padua high risk	169 (26%)	10 (21.3%)	159 (26.4%)	0.443	10 (21.3%)	32 (18.9%)	0.720
Caprini very low and low risk	3	0	3	> 0.999			–
Caprini moderate risk	49 (7.5%)	3 (6.4%)	46 (7.6%)	> 0.999	3 (6.4%)	8 (1.3%)	0.708
Caprini high risk	337 (51.8%)	32 (68.0%)	305 (50.6%)	0.021	32 (68.0%)	115 (68.0%)	0.096
Wells low-to-moderate risk	58 (8.9%)	1 (2.1%)	57 (9.5%)	0.110	1 (2.1%)	10 (5.9%)	0.463
Wells high risk	1 (0.2%)	0	1 (0.2%)	> 0.999			–
Frequency of DVT risk assessment							
Within 24 h of admission	411 (63.2%)	21 (44.7%)	390 (64.7%)	0.006	21 (44.7%)	110 (65.1%)	0.011
24-48 h of admission	201 (30.9%)	22 (46.8%)	179 (29.7%)	0.064	22 (46.8%)	53 (31.3%)	0.049
When disease conditions change	33 (5.1%)	18 (38.3%)	15 (2.5%)	< 0.001	18 (38.3%)	5 (3.0%)	< 0.001
Routinely	98 (15.1%)	8 (17.0%)	90 (14.9%)	0.699	8 (17.0%)	31 (18.3%)	0.835
DVT prevention							
Basic prophylaxis	562 (86.5%)	26 (55.3%)	536 (88.9%)	< 0.001	26 (55.3%)	158 (93.5%)	< 0.001
Physical prophylaxis	469 (72.2%)	19 (40.4%)	450 (74.6%)	< 0.001	19 (40.4%)	144 (85.2%)	< 0.001
Pharmaceutical prophylaxis	169 (26.0%)	8 (17.0%)	161(26.7%)	0.145	8 (17.0%)	38 (22.5%)	0.418
Low molecular weight heparin	126 (74.6%)	5 (62.5%)	121 (75.2%)	0.631	5 (62.5%)	30 (78.9%)	0.793
Unfractionated heparin	3 (1.8%)	0	3 (1.9%)	0.737	0	2 (5.3%)	0.432
Warfarin	5 (3.0%)	1 (12.5%)	4 (24.8%)	0.562	1 (12.5%)	0	0.135
Factor Xa inhibitor	1 (0.6%)	1 (12.5%)	0	0.012	1 (12.5%)	0	0.012
Factor IIA inhibitor	1 (0.6%)	0	1 (0.6%)	0.847	0	1 (2.6%)	0.847
Antiplatelet	33 (19.5%)	1 (12.5%)	32 (19.9%)	0.209	1 (12.5%)	5 (13.2%)	0.536

*PSM* Propensity score matching, *DVT* Deep venous thrombosis

### Factors influencing DVT

The multivariable regression analysis showed that age (≥75 vs. 60-74 years old, odds ratio (OR) = 2.091, 95% confidence interval (CI): 1.308-2.846, *P* = 0.001), the use of analgesic/sedative/muscarinic drugs (OR = 2.451, 95%CI: 1.814-7.385, *P* = 0.011), D-dimer level (OR = 1.937, 95%CI: 1.511-3.063, *P* = 0.006), high Caprini risk score (OR = 2.862, 95%CI: 1.321-2.318, *P* = 0.039), basic prophylaxis (OR = 0.111, 95%CI: 0.029-0.430, *P* = 0.001), and physical prophylaxis (OR = 0.322, 95%CI: 0.109-0.954, *P* = 0.041) were independently associated with DVT (Table 3).

**Table 3:** Multivariable logistic regression analysis of factors influencing the occurrence of DVT

Variables	Before PSM			After PSM		
	OR	95% CI	*P*	OR	95% CI	*P*
Age (≥75 vs 60-74 years old)	1.705	1.034-2.618	< 0.001	2.091	1.308–2.846	0.001
COPD	1.156	1.034–1.719	0.017	1.209	0.938–1.615	0.209
Cerebrovascular diseases (including ischemic stroke)	1.534	0.964–1.735	0.296	1.390	0.810–1.631	0.076
Patients from the operating room	2.193	0.881–2.959	0.704	2.256	0.744–6.844	0.151
Admission due to surgical diseases	2.801	1.218–6.443	0.015	2.518	0.882–5.774	0.219
Analgesic/sedative/muscle relaxant drugs	3.881	1.650–9.131	0.002	2.451	1.814–7.385	0.011
D-dimer	1.957	1.523–3.093	0.001	1.937	1.511–3.063	0.006
Caprini high risk	2.930	1.416–2.081	0.060	2.862	1.321–2.318	0.039
VTE assessment within 24 h of admission	0.593	0.284–1.235	0.163	0.765	0.325–1.800	0.540
Basic prophylaxis	0.264	0.117–0.597	0.001	0.111	0.029–0.430	0.001
Physical prophylaxis	0.258	0.117–0.568	0.001	0.322	0.109–0.954	0.041

*PSM* Propensity score matching, *COPD* Chronic obstructive pulmonary disease, *APTT* Activated partial thromboplastin time, *DVT* Deep venous thrombosis

### Treatment of DVT and patients’ outcomes

In total, 30 patients were treated with anticoagulants (28 with LMWH and two with warfarin); the thrombus was resolved in six patients. One patient received antiplatelet therapy and was followed up on the day of transfer from the ICU. Two patients received anticoagulation + antiplatelet therapy, of whom one was followed up on the day of transfer from the ICU, and the thrombus was resolved. Besides, two patients underwent interventional procedures, while the thrombus was not still resolved. In addition, 12 patients were followed up because they were at a high risk of bleeding, and the follow-up at the time of transfer from the ICU showed that the thrombus was still present.

There were no significant differences in the 28-day survival rate, 60-day survival rate, length of stay in ICU, total hospital stays, pulmonary embolism within 60 days, and coagulopathy between the two groups, while the DVT group had a higher incidence of bleeding compared with that in the non-DVT group (42.6% vs. 8.9%, *P* < 0.001) (Table 4).

**Table 4:** Patients’ outcomes

Outcomes	Before PSM			After PSM		
	DVT group (*n* = 47)	Non-DVT (*n* = 603)	*P*	DVT group (*n* = 47)	Non-DVT (*n* = 169)	*P*
28-day survival rate	41 (87.2%)	491 (81.4%)	0.320	42 (87.2%)	141 (83.4%)	0.318
60-day survival rate	38 (80.9%)	476 (78.9%)	0.756	38 (80.9%)	137 (81.2%)	0.765
ICU stay (days)	12.0 (5.0–26.0)	9.0 (5.0–17.0)	0.400	12.0 (5.0–26.0)	10.0 (6.0–25.0)	0.360
Hospital stay (days)	27.0 (12.0–43.0)	18.0 (10.0–30.0)	0.104	27.0 (12.0–43.0)	20.0 (10.0–43.0)	0.886
Pulmonary embolism within 60 days	0 (0%)	0 (0%)	–	0		–
Bleeding events	20 (42.6%)	40 (6.6%)	< 0.001	20 (42.6%)	15 (8.9%)	< 0.001
Coagulation disorders	3 (6.4%)	29 (4.8%)	0.498	3 (6.4%)	9 (5.3%)	0.726

*PSM* Propensity score matching, *DVT* Deep venous thrombosis, *ICU* Intensive care unit

## Discussion

Older adult patients mainly suffer from multiple comorbidities that increase VTE risk during ICU stay, while a limited number of studies have assessed the risk factors of VTE for older adult ICU patients. Therefore, the present study aimed to analyze the risk factors for DVT in older adult ICU patients. This post hoc subgroup analysis of a prospective, multicenter, observational study suggested that in ICU patients, age (> 75 years old), basic prophylaxis, and physical prophylaxis are independent protective factors for DVT, while age, the use of analgesic/sedative/muscarinic drugs, D-dimer level, and high Caprini risk score are independent risk factors for DVT.

Several previous studies and clinical trials of DVT excluded older adult patients because these patients mainly suffer from multiple comorbidities that are set as exclusion criteria. At present, the number of older adult patients in ICUs is noticeable. Besides the fact that age itself is a risk factor for DVT, multiple comorbidities mainly found in older adult patients make them a special population in which the classical factors for DVT might not be applied. The prevalence of asymptomatic DVT in patients aged > 80 years old might be as high as 33% [8, 17, 18], highlighting the need for proper screening of older adult patients to prevent DVT complications (e.g., pulmonary embolism or stroke). VTE is diagnosed in about 4.5-7.3% of older adult ICU patients [19, 20], suggesting a great number of older adult ICU patients who were not diagnosed with VTE. In the present study, the frequency of such patients was 2.3%.

Hypercoagulability, a member of Virchow’s triad, is a condition in which the hemostatic balance is tilted towards thrombus formation [1–3], and it is an important factor for DVT [21]. In older adults, increased coagulation factors are a major cause of hypercoagulability [1–3]. In the present study, D-dimer level and age were found to be associated with DVT risk. D-dimer is a well-known marker of DVT [22]. It is detectable in patients with DVT because of ongoing endogenous fibrinolysis [23]. Age is an important factor associated with increased coagulability [11].

Another member of Virchow’s triad is blood stasis [1–3]. The use of analgesic, sedative, and muscarinic drugs can increase the DVT risk because neuromuscular blockade may inevitably induce immobility, which is a risk factor for DVT [24]. Although the use of such drugs is often necessary to allow mechanical ventilation and to prevent pain, their use may increase the risk of VTE [1–3]. Thus, older adult patients who must receive analgesic, sedative, or muscarinic drugs need to be closely monitored for DVT. Additionally, since immobility is a risk factor for DVT, such drugs also increase the DVT risk in younger patients [24]. Whether the influence of the use of such drugs on the older adult is similar to or greater than on younger adults remains to be elucidated.

The Caprini risk assessment is a validated and reliable tool for VTE [25], but it is not specific to older adult patients. In addition, the use of prophylaxis decreases the risk of VTE [26, 27]. Therefore, the association of these factors with VTE risk is obvious. Moreover, drug prophylaxis is well recognized to decrease VTE risk [25, 27–29]; however, in the present study, drug prevention was not associated with the decreased risk of VTE in older adult patients. This difference between the general ICU population and the older adult ICU population could be because of the use of different drugs, various conditions leading to ICU admission, comorbidities, and different drugs used for older adult patients. The optimal VTE prophylaxis in older adult patients might be different from the general population. Studies specifically examining the efficacy of VTE drug prophylaxis in older adults versus younger patients are necessary to refine the prophylactic strategies against DVT in older adult ICU patients.

Wang et al. showed that hypertension, cancer or systemic cancer treatments, diabetes, coronary heart disease, heart failure, respiratory failure, acute myocardial infarction, and ischemic stroke were associated with the risk of VTE in older adult ICU patients [28]. Chen et al. [12] indicated that gender (male), bedridden for > 72 h, pneumonia, history of DVT, diabetes, coronary heart disease, glucocorticoids, PaO_2_, mechanical ventilation, CRRT, hemoglobin level, PT, INR, and D-dimer level were risk factors for VTE in critically ill older adult patients. The discrepancies in risk factors among studies might be due to the exact ICU population, local clinical practice, variables being examined, and different definitions used. A strength of the present study was the relatively large sample size from 54 hospitals, covering a large proportion of Zhejiang Province. The inclusion of multiple hospitals might decrease the impact of the differences on local practice. In addition, in China, the application of the official guidelines for VTE management is strongly emphasized, decreasing the likelihood of significant differences in local practice among the hospitals. Compared with patients without DVT, patients with DVT have an increased risk of bleeding. The reason is that patients with DVT may be high-risk patients or have already developed DVT and are receiving anticoagulation prophylaxis or treatment, resulting in an increased risk of bleeding.

The results of a previous study by the authors [13] showed that D-dimer levels, basic prophylaxis, and physical prophylaxis were independently associated with DVT in ICU patients. The subgroup analysis of middle-aged and older adult patients in the present study showed that age, the use of analgesic/sedative/muscarinic drugs, D-dimer levels, and high Caprini risk score were independently associated with DVT. Therefore, the results suggest that 1) the older the patients, the more likely DVT can occur, and DVT prevention and early screening should be strengthened. 2) If the D-dimer levels of older adult patients with severe diseases were increased after using analgesic/sedative/muscarinic drugs, early DVT screening should be conducted to strengthen prevention. 3) In the original study, it was found in all critically ill patients that basic prophylaxis (OR = 0.092, 95%CI: 0.016-0.536, *P* = 0.008) and physical prophylaxis (OR = 0.159, 95%CI: 0.038-0.674, *P* = 0.013) were protective factors. In the present study, basic prophylaxis (OR = 0.111, 95%CI: 0.029-0.430, *P* = 0.001) and physical prophylaxis (OR = 0.322, 95%CI: 0.109-0.954, *p* = 0.041) were also protective factors in older adults, but their effect appeared smaller than in the general population of ICU patients. These results might suggest that basic prophylaxis and physical prophylaxis have smaller preventive effects on DVT in older adult patients, and they might need to be strengthened or combined early with anticoagulant therapy. Still, future studies should directly compare the effect of DVT prophylaxis between adults and older adults.

This study has some limitations. First, the follow-up period was short (i.e., limited to the hospital stay), and DVT events were examined only during hospitalization. Second, as an observational study, the definite effects of DVT prophylaxis could not be obtained, and future randomized controlled trials are needed to analyze the efficacy and safety of prophylaxis. Third, only analgesic/sedative/muscarinic drugs were analyzed. Although several other drugs can influence the risk of DVT, their diversity was too large and their individual frequency too small to be included in the analyses. On the other hand, analgesic/sedative/muscarinic drugs are commonly used in the ICU. Fourth, this study was conducted in Zhejiang Province, and the generalizability of the results is unknown. Finally, as it was an observational study based on the routine practice of each participating hospital, no unified method of risk assessment was used. Different centers used different scales and performed reassessments at different frequencies.

In conclusion, in critically ill older adult patients, age (≥75 years old), basic prophylaxis, and physical prophylaxis were found as independent protective factors for DVT. In contrast, age, use of analgesic, sedative, and muscarinic drugs, D-dimer level, and high Caprini risk score were noted as independent risk factors for DVT. Additional studies are necessary to examine and compare the specific risk factors for DVT between older adults and younger patients in the ICU.

## Data Availability

All data generated or analyzed during this study are included in this published article.

## Abbreviations

VTE: Venous thromboembolism
ICU: Intensive care unit
DVT: Deep venous thrombosis
CRRT: Continuous renal replacement therapy
PT: Prothrombin time
INR: International normalized ratio
UFH: Unfractionated heparin
LMWH: Low-molecular-weight heparin
IPC: Intermittent pneumatic compression
GCS: Graduated compression stockings
APTT: Activated partial thromboplastin time
APACHE II: Acute Physiology and Chronic Health Evaluation II
PSM: Propensity score matching
COPD: Chronic obstructive pulmonary disease
CVD: Cerebrovascular disease
